# Confirmatory Factor Analysis of the Short Form McGill Pain Questionnaire With Burn Patients

**Published:** 2008-12-01

**Authors:** Shawn T. Mason, Lisa L. Arceneaux, William Abouhassan, Dean Lauterbach, Caryn Seebach, James A. Fauerbach

**Affiliations:** ^a^ Department of Psychiatry & Behavioral Sciences, Johns Hopkins University School of Medicine, Baltimore, MD; ^b^ Johns Hopkins Burn Center, Johns Hopkins Bayview Medical Center, Baltimore, MD; ^c^ Department of Surgery, University of Texas Medical Branch, Baltimore, MD; ^d^ Shriners Hospital for Children, Galveston, TX; ^e^ Department of Psychology, Eastern Michigan University, Ypsilanti, MI; ^f^ Department of Psychology, Loyola University, Baltimore, MD

## Abstract

The Short Form McGill Pain Questionnaire (SF-MPQ) is an abbreviated version of McGill Pain Questionnaire (MPQ) developed for pragmatic reasons to improve the clinical utility of the MPQ. Although the SF-MPQ has been used in more than 250 published studies, few studies have examined the core constructs it measures. The objective of this study was to evaluate in a sample with burn injuries whether the factor structure of the SF-MPQ is consistent with the theoretic pain constructs it purports to measure. Participants (*n* = 338) met American Burn Association's criteria for major burn injury and had a mean total body surface area burned of 14%. They were mostly male (70.1%) and Caucasian (63.4%) with an average age of 41.25 years. There were 2 primary findings. First, confirmatory factor analysis yielded fit index values demonstrating viability of a 2-factor, oblique, solution composed of sensory and affective latent constructs. These findings were consistent with previous work and the theoretic constructs. Second, results from a relatively new model consisting of 8 SF-MPQ items demonstrated potential viability for measuring similar constructs.

Pain is ubiquitous in the burn intensive care setting, and for some patients it is a lifelong burden.^1^ Burn pain results not only from the inflammatory pain of the acute injury that has multiple mechanisms (eg, thermal, flame, electrical, or scald) but also from daily burn wound dressing changes, physical therapy, and neuropathic pain associated with skin grafts and scar contractures. Anecdotally, patients describe *burn pain* as the worst pain they have ever experienced, and research has shown deleterious long-term outcomes associated with burn pain.^2^ As such, clinical and research efforts to measure and understand pain are important. This study used a fundamental psychometric measurement technique called confirmatory factor analysis (CFA) to investigate the factor structure of the Short Form McGill Pain Questionnaire (SF-MPQ). The CFA method describes components of the measure itself and the construct that it purports to assess.

## FACTOR STRUCTURE

Factors are subcategories, or dimensions, of a more general topic (eg, sensory pain is a dimension of total pain phenomena). Factor analysis reflects covariance between multiple variables simultaneously and identifies major clusters. Hence, *relatedness* among the variables can be demonstrated. Conceptually, factors represent hypothesized *causal mechanisms* within a given domain or disorder.^3^ Confirmatory factor analysis is a structural equation modeling method used to test the viability of hypothesized models.

## THE SHORT FORM McGILL PAIN QUESTIONNAIRE

The SF-MPQ is an abbreviated version of the MPQ and was designed by Melzack^4^ to provide a brief, but useful measure of pain. Melzack proposed that the SF-MPQ consisted of 2 independent factors. One was referred to as *sensory*, which described the nociceptive pain experience of the individual, and the other was referred to as *affective*, which described the emotional impact of the nociceptive pain experience. This will hereafter be referred to as the Melzack model.

Five studies have examined the factor structure of the SF-MPQ. Three of these studies examined the factor structure of the SF-MPQ, using only exploratory factor analysis (EFA),^5^–^7^ while one study assessed factor structure, using CFA alone,^8^ and another study used both EFA and CFA.^9^ Typically, EFA studies are used for scale development or theory building. Given that the SF-MPQ was designed to measure 2 hypothesized factors, CFA is an appropriate approach to test the viability of this model. However, alternative and competing models can also be tested to examine which model best describes the data. Thus, both EFA and CFA studies investigating the factorial validity of the SF-MPQ are reviewed next.

## EXPLORATORY FACTOR ANALYSIS STUDIES

Burckhardt and Bjelle^5^ reported a 3-factor solution for a Swedish version of the SF-MPQ. Participants consisted of 100 women reporting a history of either fibromyalgia or rheumatoid arthritis, which both have a prominent component of pain (mean age-45 years). The fibromyalgia (*n* = 50) group reported average time since disease onset of 8 years. The rheumatoid arthritis (*n* = 50) group reported average time since disease onset of 15 years. Exploratory factor analysis was conducted on data from the entire sample using principal components extraction with varimax rotation. Findings suggested that the sensory factor may be composed of 2 separate dimensions reflecting acute-sensory and chronic-sensory features. However, their results were not definitive, as many cross loadings (loadings > 0.30 on more than 1 factor) were observed. That is, items in their study showed significant relationships with more than 1 factor, thus indicating a highly intercorrelated solution making it very unclear what is actually being measured. This occurred on 10 of the 15 items. Interestingly, the affective factor remained largely stable. Hereafter, this is referred to as the Burckhardt 3-factor model.

Beattie et al^9^ reported different results from a sample of 187 patients undergoing lumbar magnetic resonance imaging (MRI) to diagnose chronic lower back pain. The sample was primarily female (52%) with a mean age of 48 years.^9^ Most participants (64%) reported the co-occurrence of distal pain and 36% reported only lower back pain. EFA procedures included a principal axis extraction and varimax rotation. A 2-factor solution was reported, and results were largely consistent with one affective factor and one sensory factor. However, problematically, not all loadings met criteria for item retention (ie, item loadings > 0.50 without cross loadings). The authors proposed 2 factors called “sensory” and “sensory-affective” that together consisted of only 8 of the original 15 items. The 8 items showing unique contributions to the EFA solution were referred to as the Modified Short Form–MPQ (MSF-MPQ). Factor 1 items were “Shooting, Sharp, and Stabbing.” Factor 2 items were “Splitting, Fearful, Heavy, Sickening, and Punishing-Cruel.” Hereafter, this model will be referred to as the 2-factor MSF-MPQ model. The CFA cross-validation analysis is described later.

Two studies have investigated the cross-cultural validity of the SF-MPQ by comparing ethnically diverse samples. Cassisi et al^6^ used 2 samples: one was African American (*n* = 97) and the other was European American (*n* = 392). Altogether, participants had a mean age of 50 years while the African American sample reported higher rates of divorce, separation, or being widowed, as well as reporting lower education. Across samples, pain was primarily due to lower back and lower limb pain (67%) and upper shoulder and upper limb pain (12%). EFA procedures were conducted using principal axis extraction and an oblique (oblimin) rotation. In the African American sample, 5 factors accounted for 61% of the total variance. Of note, the Visual Analog Scale (VAS) score was included in these analyses whereas previous studies omitted this component from factor structure analysis. Factor-item correspondence was as follows: Factor 1 items were “Punish, Fearful, Sickening, and Splitting,” and the factor 2 item was “Throbbing,” the factor 3 item was pain intensity (ie, the VAS), factor 4 items were “Gnawing, Heavy, Cramping, Tiring, and Sharp,” and factor 5 items were “Stabbing, Tender, Aching, and Hot.” The “Shooting” item did not load significantly on any factor. Hereafter, this model is referred to as Cassisi A.

The European American sample studied by Cassisi et al^6^ produced a 4-factor solution that accounted for 52% of the total variance. Factor-item correspondence was as follows: Factor 1 items were “Heavy, Aching, Tiring, Gnawing”, and the VAS, factor 2 items were “Stabbing, Shooting, and Sharp,” factor 3 items were “Fearful, Punish, Sickening, and Splitting,” and factor 4 items were “Tender, Hot, Throbbing, and Cramping.” This model is hereafter referred to as the Cassisi B model. For both samples, the authors noted difficulty in labeling the factors to capture their conceptual essence. This indicates that the items did not settle on factors in an interpretable pattern and greatly reduces the utility of their model.

It should be noted that the current study could not directly test the relative viability of the 2-factor structures identified by Cassisi et al (Cassisi A and Cassisi B). This is because the factor structure results from the African American sample (Cassisi A) could not be tested by CFA in their exact form since 2 of the reported factors consisted of single-item components, one of which was the VAS. Factor structure results from the European American sample (Cassisi B) also included the VAS. However, each factor contained 3 or more items, even without the VAS. To provide some means to compare the present study with prior work in this area, slight modifications were made to both the Cassisi A and B models providing a gross measure of evaluating their correspondence. Details follow in the “Data Analysis” section.

In a second investigation of cross-cultural validity, EFA was conducted on the SF-MPQ using a sample of Asian American patients with cancer.^7^ The sample consisted of 119 predominantly female (82%) patients with breast cancer (65%) who had undergone chemotherapy (78%). Most were college educated (68%) and were not working (70%), and the mean age was 52.2 years. Almost all participants were foreign born (96%) but reported fluency in written and spoken English. Exploratory factor analysis was conducted using principal components analysis and a varimax rotation. The solution accounted for 65.7% of the total variance and produced 2 factors. However, item-factor assignment was inconsistent with Melzack's model and 2 of the items (eg, “Aching and Sickening”) showed significant relationships with both factors. The first factor consisted of the following items: Punishing, Sharp, Shooting, Stabbing, Splitting, Heavy, Fearful, Tiring, and Throbbing. The second factor consisted of the following items: Tender, Hot-Burning, Cramping, and Gnawing. However, the authors did not identify conceptual relationships captured by the identified factors, and the sample size was smaller than recommended; thus, these results might demonstrate subtle language interpretation differences when assessing pain. Hereafter, this is referred to as the Shin 2-factor model.

## CONFIRMATORY FACTOR ANALYSIS STUDIES

Wright et al^8^ conducted the first CFA study of the SF-MPQ and found support for a 2-factor model consisting of sensory and affective dimensions. The sample consisted of 188, mostly male (59%), participants with chronic low back pain, some suffering for many years (eg, range = 3–432 months). The Melzack model was not robustly supported by the results. Fit index values fell short of minimum criteria for model fit. For example, the Comparative Fit Index (CFI) was 0.86 and the Adjusted Goodness of Fit Index (AGIF) was 0.81, and both are substantially below the accepted 0.90 criterion.^10^ Model alterations were required to obtain adequate fit index values. For example, one item was moved from the sensory to the affective factor (eg, gnawing), and 4 sets of error terms were set to correlate. No substantive basis for the changes was provided. After model specifications were changed, fit indices met criteria. Although the results were not entirely supportive of the model, this study demonstrated the potential for the 2-factor Melzack model with minor deviations. Hereafter, this model is referred to as the Wright 2-factor model.

After providing results of the EFA described above, Beattie et al^9^ conducted a CFA, using the split sample of 186 lumbar MRI patients for cross-validation and then the entire sample of 373. There were no significant differences for age, gender, and pain intensity between the EFA and CFA samples. Differences in CFA results between the 186 and 373 participant samples were also generally negligible. In the study presented here, 4 models were tested: (1) the original Melzack model, (2) the Wright et al model, (3) the model derived from the Beattie et al EFA results described above (MSF-MPQ), and, lastly, (4) a single-factor model. The MSF-MPQ produced the best fitting index values for both the split and total samples. Less distinguishable were the single-factor and Wright models, which showed adequate fit. The Melzack model values were well below accepted criteria.

The objective of this study was to examine the SF-MPQ factor structure in a sample of burn patients at the time of discharge from acute hospitalization. Each of the models presented in previous EFA and CFA studies of the SF-MPQ was tested (eg, Melzack 2-factor, Wright 2-factor, Cassisi A, Cassisi B, Shin 2-factor, Burckhardt 3-factor, and 2-factor MSF-MPQ). This represents a novel contribution to the literature in that there are currently no CFA studies using SF-MPQ data from burn patients.

## METHODS

### Study population

Participants were adult patients admitted to the Johns Hopkins Burn Center from January 1998 through June 2006 for treatment of a major burn injury who consented to this 2-year prospective study of functional outcomes. Eligibility criteria were ≥18 years of age, able to provide consent, and met American Burn Association criteria for major burn injury. ABA major burn criteria are the presence of any of the following: 5% total body surface area (TBSA) Full-thickness burn; 10% TBSA partial thickness burn for pediatric or elderly; 20% TBSA partial thickness burn for adults; burns to vulnerable cosmetic or functional areas such as face, hands, feet, perineum, or across joints, or that involve inhalation injury or circumferential burns; or burns involving electrical or chemical mechanisms. The Johns Hopkins School of Medicine institutional review board approved this study.

Of the 338 participants, most were male (70.1%), Caucasian American (63.4%), married or living together (49.2%), and employed (74.1%) with high school education or less (68.5%). The average age was 41.25 years (SD = 15.23). The average TBSA burned was 14.1% (SD = 12.97). Approximately 78.7% required 1 or more operations. Hand injuries (60.2%), skin contractures affecting range of motion in 1 or more joints at discharge (73.7%), and burns involving the face, head, or neck (42.3%) were common while amputations were not (1.7%). Preexisting physical and psychiatric comorbidity was common; for example, 11.9% had sought mental health treatment in the year before the injury.

The SF-MPQ is a 15-item self-report scale derived from the McGill Pain Questionnaire.^11^ The SF-MPQ contains 3 components. The Pain Rating Index (PRI) consists of 15 representative words that are rated on a 4-point Likert-type rating scale ranging from 0 (*none*) to 3 (*severe*). It includes 11 sensory (eg, tender) and 4 affective (eg, sickening) items. In addition, there are 2 items measuring pain intensity. Overall pain is assessed on a numeric analogue scale (NAS), where ratings are made on a 10-cm visual line that approximates ratings between 0 (*no pain*) and 10 (*unbearable pain*). The Present Pain Intensity (PPI) scale is a verbal analogue scale (VAS) with values from 0 (*no pain*) to 5 (*excruciating*). The NAS and the PPI were not used in the current evaluation of SF-MPQ factor structure because they were not identified in any previous MPQ or SF-MPQ factor analysis studies as contributing to the dimensional characteristics. Temporal stability of the SF-MPQ has been shown to be moderate to high—retest reliability coefficients ranging from 0.45 to 0.96. Internal consistency values, as measured by the Cronbach alpha, range from .73 to .94 for subscales and total severity scores.^5^,^7^,^12^

### Procedures

This study was conducted as part of a larger, multisite, longitudinal outcomes study following major burn injury. Study measures were administered by a trained and supervised research assistant approximately 1 to 3 days prior to discharge from the index hospitalization, and follow-up measures were completed by established protocol at 6, 12, and 24 months postdischarge. Discharge assessment data are examined in this study.

### Data analysis

Data were examined for characteristics (eg, missing data, zero inflation, and normality) using SPSS 16.0 and Mplus 4.21 (Muthen & Muthen, 2007). Skew and kurtosis indicators showed minimal deviation from a normal distribution and are included with descriptive statistics. Zero inflation (eg, individuals reporting *no* pain) was minimal including only 1.8% of the sample. Missing values were observed on 9 individual assessments that demonstrated no common pattern. Each pattern was unique to 2 or fewer individuals. Missing data were handled in the analyses using Robust Maximum Likelihood Estimation. Items were treated as continuous indicators of latent constructs in all CFAs.

CFAs were conducted to evaluate the 8 measurement models described above for fit with the burn patient data. They were all first-order models and included: (1) a single factor model that represented pain as a unitary construct; (2) a 2-factor Melzack model with sensory and affective components; (3) a modified 2-factor Melzack model of Wright et al; (4) a 2-factor model of Shin et al; (5) Burckhardt and Bjelle's 3-factor model derived; (6) Cassisi's African American sample 3-factor model without the VAS and single-item factors (Cassisi A); (7) Cassisi's European American sample 4-factor model without the VAS (Cassisi B); and (8) an 8-item, 2-factor MSF-MPQ of Beattie et al. The Wright model was originally specified to allow 4 sets of error variances to correlate and to move 1 item from the sensory to the affective factor. Error variances were not allowed to correlate because of the absence of substantive rationale (eg, method variance). However, reassigning 1 item to the alternate factor to test the model was deemed reasonable in the face of potential semantic variation. In the Shin model, item assignment was consistent with EFA results and the 2 cross loading items were omitted (eg, Aching and Sickening). Please see Table 1 for item assignment to each model.

#### Indices of model fit

Indices used to evaluate model fit in CFA include chi-square (χ^2^), the standardized root-mean-square residual (SRMR), the root-mean-square error of approximation (RMSEA),^13^ the CFI,^14^ the Tucker-Lewis Index (TLI),^15^ the Akaike Information Criterion (AIC),^16^ and the Bayesian Information Criterion (BIC).^17^ In chi-square analyses, the model with the lowest tabled values and the fewest degrees of freedom (ie, most parsimonious) is desired. The SRMR, RMSEA, TLI, and CFI produce values that generally range between 0 and 1. For SRMR and RMSEA, lower values are desired as opposed to CFI and TLI, where higher values indicate *fit*. The AIC and BIC are relative indices, where lower values are desired when comparing competing models. Many of the guidelines for interpreting each of these indices were compiled by Brown.^18^ If CFI and TLI values were between 0.90 and 0.95, they indicated adequate fit. Values above 0.95 indicated good fit and values below 0.90 indicated poor fit.^10^,^14^ For the RMSEA, values above 1.0 should be rejected and those below 0.06 indicate good fit. For the SMSR, values less than 0.08 indicate adequate fit and values below 0.05 indicate good fit.^10^,^19^

## RESULTS

### Descriptive statistics

Internal consistency reliability (the Cronbach alpha) was .86. On the 0 to 3 scale, item means ranged from 0.61 (Splitting and Fearful) to 1.99 (Tender). The VAS mean was 4.40 and the PPT mean was 3.01. The mean total score from the 15 SF-MPQ items was 15.11 (SD = 11.61). Please see Table 2 for details.

### Confirmatory factor analysis

The viability of 8 measurement models was tested. The 2-factor MSF-MPQ model proved the best fitting model according to relative and absolute indices (Table 3, last row). Lower relative values for chi-square, SRMR, RMSEA, AIC, and BIC, and higher CFI and TLI values indicated better model fit. Values of SRMR, RMSEA, CFI, and TLI met criteria for adequate model fit. There is no specific difference test used to compare nonnested CFA models. Fit index values can only evaluate each model independently against published criteria. As such, theoretical bases and parsimony were used to compare nonnested models.

Two models provided adequate fit to the data but were less robust relative to the 2-factor MSF-MPQ. The 2-factor Melzack and 3-factor Burckhardt models were indistinguishable according to values (eg, CFI = 0.91 and TLI = 0.90).

Several of the models evaluated provided poor fit to the data. The single-factor model (Table 3, first row) did not meet most fit index criteria. The 2-factor Wright model did not meet the 0.90 criteria for model fit for the TLI (TLI = 0.88). Results from the 2-factor Shin and both 3- and 4-factor Cassisi models could not be reported. This was because analyses testing these models produced a nonpositive definite matrix, indicating linear dependency or a correlation greater than 1 between latent variables.

**Figure 1:**
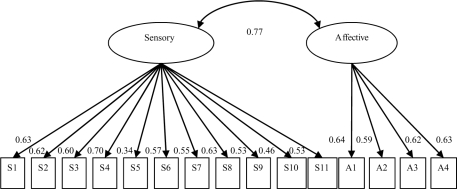
Melzack 2-factor model illustration with standardized factor covariance and loadings.

**Figure 2:**
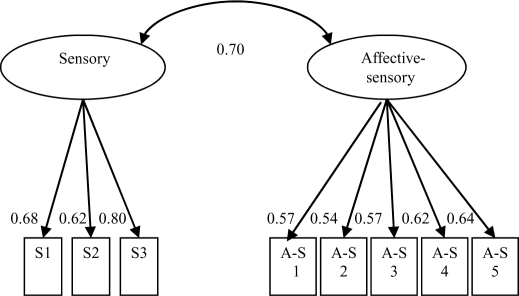
MSF-MPQ 2-factor model illustration with standardized factor covariance and loadings.

SF-MPQ standardized factor loadings for the 2-factor Melzack model ranged from 0.34 to 0.70 for the sensory factor and from 0.59 to 0.64 for the affective factor, and the covariance between factors was 0.77. Standardized factor loadings for the 2-factor MSF-MPQ model ranged from 0.62 to 0.80 for the sensory factor and from 0.54 to 0.64 for the affective-sensory factor, and the covariance between factors was 0.70. See Figures 1 and 2 for Melzack and MSF-MPQ models, respectively, for factor loadings and covariance.

## DISCUSSION

This is the first study to use a population of burn victims to investigate the factor structure of a pain measurement tool commonly used in clinical and research settings. The primary aim of this study was to test Melzack's contention that the SF-MPQ consists of 2 factors. Second, the study aimed to test alternative models proposed from other research findings. This study did confirm the viability of the 2-factor structure proposed by Melzack for the SF-MPQ among burn patients. However, the 2-factor MSF-MPQ model was the best fitting model in this sample. Fit index values fell in the “good fit” range when compared with the Melzack model, in which index values fell into the “adequate fit” range. The Melzack model was preferred over all other models except for the MSF-MPQ because its 2-factor solution was more parsimonious and established than those of other models.

An interesting facet of this study includes the evaluation of models derived from various ethnic samples. The EFA models produced by data from African American and Asian American samples were not viable in CFA due to linear dependency. Notably, the Cassisi et al European American EFA model was not viable either. While EFA models are not always viable in CFA, this finding suggests meaningful differences in the use of pain reporting language within fluent English-speaking groups with different ethnic backgrounds.

When considering preference between the Melzack and MSF-MPQ models, some issues must be addressed. First, the MSF-MPQ consists of 8 items, while the SF-MPQ consists of 15 items. Although it is possible that the MSF-MPQ is a superior measure, the relative lack of studies investigating this model weakens this argument. Second, important items may be omitted in the MSF-MPQ. For example, in this sample of burn patients, the “Tender” item has the highest individual ratings and is not included in the MSF-MPQ. Although decrements in model fit were not observed here, other important types of pain may not be captured by the truncated 8-item scale.

Future SF-MPQ studies should further investigate the viability of the MSF-MPQ, its factor structure, item function, sensitivity and specificity to pain, and ability to detect change over time and with treatment. Strengths of this study include the large sample size, relatively high degree of pain reporting, and the clear identification of the acute pain source (eg, burn injury). The nature of the pain may well be both a strength and limitation of this study. The acute and severe nature of the pain provides a narrow range for investigation. Alternatively, acute burn pain reporting may not generalize to some pain populations. Other limitations of this study include our inability to directly compare several competing models. Nonnested factor models require additional consideration for comparison (eg, theory, parsimony, or previous findings). Also, there was no information regarding previous pain history or comorbid pain conditions. Future studies should consider testing the MSF-MPQ model when evaluating the SF-MPQ in other pain populations. Until findings suggest otherwise, the SF-MPQ should continue to be used in its entirety.

## ACKNOWLEDGMENTS

This study was supported by the Burn Injury and Rehabilitation Model Systems grant funded by the National Institute on Disability and Rehabilitation Research in the Office of Special Education and Rehabilitative Services in the US Department of Education. NIDRR Grant H133A070045.

## Figures and Tables

**Table 1:** Model specifications and item assignment

		**Models**							
**Item**		Single	Melzack	Cassisi A	Cassisi B	Wright	Shin	Burckhardt	MPQ
1	Throbbing	M	S	X	C4	S	S1	S-A	X
2	Shooting	M	S	X	C2	S	S1	S-A	S
3	Stabbing	M	S	C3	C2	S	S1	S-A	S
4	Sharp	M	S	C2	C2	S	S1	S-A	S
5	Cramping	M	S	C2	C4	S	S2	S-A	X
6	Gnawing	M	S	C2	C1	A	S2	S-C	X
7	Hot burning	M	S	C3	C4	S	S2	S-A	X
8	Aching	M	S	C3	C1	S	X	S-C	X
9	Heavy	M	S	C2	C1	S	S1	S-C	A-S
10	Tender	M	S	C3	C4	S	S2	S-C	X
11	Splitting	M	S	C1	C3	S	S1	S-A	A-S
12	Tiring-exhausting	M	A	C2	C1	A	S1	A	X
13	Sickening	M	A	C1	C3	A	X	A	A-S
14	Fearful	M	A	C1	C3	A	S1	A	A-S
15	Punishing–cruel	M	A	C1	C3	A	S1	A	A-S

M indicates SF-MPQ; S, sensory; A, affective; Cassissi A, African American sample; Cassisi B, European American sample; C1, Cassisi factor 1; C2, Cassisi factor 2; C3, Cassisi factor 3; C4, Cassisi factor 4; S1, shin factor 1; S2, shin factor 2; S-A, sensory acute; S-C, sensory chronic; X, not used; and A-S, affective sensory.

**Table 2:** SF-MPQ item and total severity score means and standard deviations

SF-MPQ item	Item means (SD)	Skew	Kurtosis
Throbbing	1.36 (1.08)	0.04	−1.31
Shooting	1.01 (1.08)	0.55	−1.10
Stabbing	0.74 (1.01)	1.02	−0.33
Sharp	1.36 (1.13)	0.11	−1.39
Cramping	0.67 (0.93)	1.15	0.14
Gnawing	0.80 (1.08)	0.93	−0.65
Hot burning	1.33 (1.19)	0.18	−1.46
Aching	1.50 (1.07)	−0.10	−1.24
Heavy	0.82 (1.10)	0.96	−0.57
Tender	1.99 (1.03)	−0.59	−0.82
Splitting	0.61 (1.00)	1.33	0.31
Tiring-exhausting	1.18 (1.12)	0.35	−1.29
Sickening	0.62 (0.98)	1.39	0.62
Fearful	0.61 (1.00)	1.39	0.49
Punishing–cruel	0.56 (1.02)	1.56	0.89
Visual Analog Scale	4.40 (2.62)	0.04	−1.31
PPI	3.01 (1.12)	0.55	−1.10
SF-MPQ 15-item TS*	15.11 (11.61)	1.02	−0.33

* SF-MPQ total score is composed of the first 15 items and excludes the Visual Analog Scale and the Present Pain Intensity items.

**Table 3:** Confirmatory factor analysis fit index values

Model	df	χ^2^	SRMR	RMSEA	AIC	BIC	CFI	TLI
Single-factor	90	223.82	0.06	0.06	13738	13910	0.87	0.85
Melzack	89	184.20	0.05	0.06	13369	13866	0.91	0.90
Wright	89	197.25	0.05	0.06	13706	13882	0.90	0.88
Burckhardt	87	177.22	0.05	0.06	13685	13869	0.91	0.90
MSF-MPQ	19	32.90	0.04	0.05	7286	7382	0.97	0.95
